# Insights into Tear Film Stability from Babies and Young Adults: A Study of Human Meibum Lipid Conformation and Rheology

**DOI:** 10.3390/ijms19113502

**Published:** 2018-11-07

**Authors:** Poonam Mudgil, Douglas Borchman, Aparna Ramasubramanian

**Affiliations:** 1 School of Medicine, Western Sydney University, Locked Bag 1797, Penrith, NSW 2751, Australia; p.mudgil@westernsydney.edu.au; 2 Department of Ophthalmology and Visual Sciences, University of Louisville, Louisville, KY 40202, USA; aparna.ramasubramanian@louisville.edu

**Keywords:** Langmuir trough, meibum, spectroscopy, tear film, tear film lipid layer

## Abstract

Babies have the most stable tears and people with dry eye have the least stable tears. Meibum may contribute to tear film stability, so in this study, the hydrocarbon chain conformation and rheology of meibum from babies was studied for the first time. Infrared spectroscopy was used to measure lipid phase transitions. Rheology was measured using Langmuir film technology. Meibum from 25 donors 1 to 13 years old was compared with meibum from 18 donors 13 to 25 years old. The phase transition temperature and lipid order (stiffness) increased with increasing age from 1 to 25 years. The increase in meibum lipid order from 1 to 25 years of age may contribute to the instability of the tear film with age and contribute to films with a higher reciprocal compressibility modulus that are not as compressible and not as viscoelastic. Changes in the lipid phase transition parameters of meibum lipid with dry eye are an exacerbation of the changes observed with age. The lower reciprocal compressibility moduli of meibum films from children and babies compared with meibum from adults reiterates higher stability in their films which spread better, resist deformation, and facilitates their ability to be quickly restored after blinking.

## 1. Introduction

Among many functions, tears keep the cornea hydrated. The tear film on the surface of the cornea is spread upon blinking. If the eye were to remain open, the tear film “breaks up” resulting in dry regions on the surface of the cornea. Chemical and temperature receptors on the surface of the cornea signal the brain to cause a blink spontaneously before drying occurs [1]. Infants have an unusually stable tear film [2,3,4,5,6,7,8,9,10,11,12,13,14,15,16]. The spontaneous blink rate of infants increases by 250% from birth to the first year of age, from about two to five blinks per minute [4]. The blink rate only doubles to about 10 blinks per minute from one year to 25 years of age, and only increases by 10% by 60 years of age [3,4,5,9,10,11,12,13,14,15,16,17,18]. A similar trend is observed for tear break up time measured by fluorescence spectroscopy [2,6,7,8]. Thus, the major changes in tear film stability occur from birth to 25 years of age. Tear film stability could be related to meibum composition and tear film lipid structure. Compared with adults, infants have less meibum on the lid reservoir [19], meibum that is more saturated [20,21,22], more ordered [22,23], and contains more proteins [20]. Saturation is the relative amount of single C-C bonds whereas unsaturation is the relative amount of C=C double bonds. When lipids are stiff and ordered like butter or lard, the lipid chain carbons are arranged in a *trans* conformation and the hydrocarbon chains are straight enabling them to pack closely together maximizing Van der Waal’s interactions. When lipids are fluid like olive oil, *gauche* rotamers cause bends in the hydrocarbon chains minimizing how close the chains can pack. 

We found that saturation increased the phase transition temperature of human meibum by over 20 °C, a relatively high amount [24,25]. The phase transition temperature is the temperature at which half of the lipid molecules undergo a change from an ordered gel phase to a disordered liquid crystalline phase. By breaking lipid–lipid interactions with increasing temperature, the strength of the lipid–lipid interactions can be calculated. With increasing temperature, many lipids undergo a phase transition from an ordered phase called the gel phase to a disordered phase called the liquid crystalline phase. The amount of casual lipid on the eye lid decreases with increasing meibum lipid order, phase transition temperature, and age [26]. Surface pressure area studies suggest that a saturated meibum film is quite molecularly ordered (stiff molecular arrangement) and elastic (molecules are able to rearrange during compression and expansion) compared with native meibum films which are more fluid.

In this in vitro study, using Langmuir trough technology, we tested if the surface properties of meibum from infants were different from that of adults. We used infrared spectroscopy to measure phase transition parameters to assess meibum lipid–lipid interaction strength and the conformation of meibum from infants and children 1 to 12 years-old and adolescents 13 to 25 years old.

## 2. Results

Donors were divided into two cohorts, young, <13 years-old (*Cy*) and older, and 13 to 25 years-old (*Co*). The demographics of the meibum donors are provided in Table 1. The contribution of the one Asian in the *Cy* cohort and three three Asians in the *Co* cohort was negligible to the phase transition parameters measured as removing them changed the reported average Tm (temperature of melting), order, and cooperativity by only 0.05 ± 1.9%. Three samples from Caucasians representing *Cy* were used for the Langmuir trough study: a female, 8 years 5 months-old and males 1 year 8 months-old and 6 months-old.

### 2.1. Phase Transition Parameters by Infrared Spectroscopy

**Figure 1:**
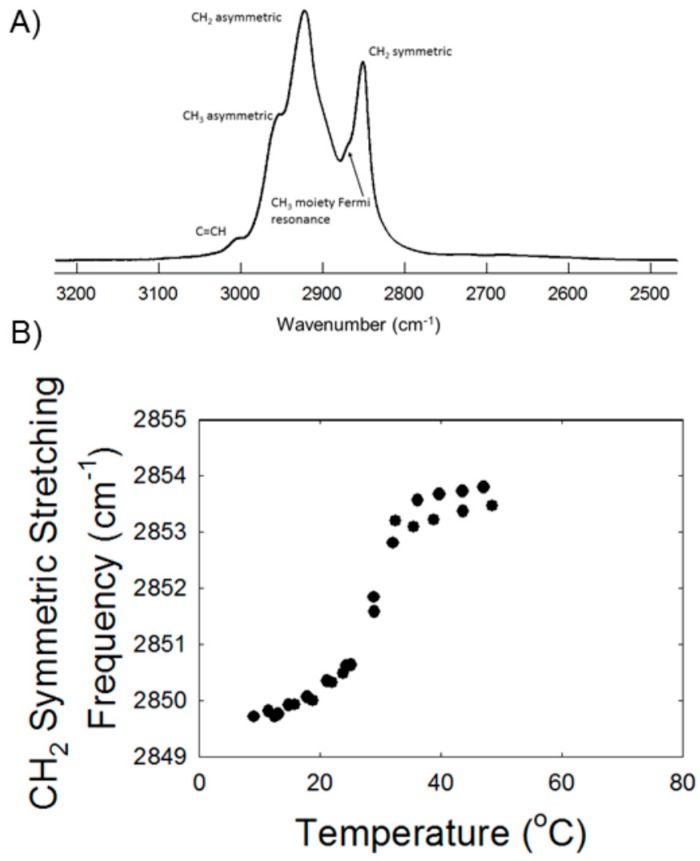
(**A**) Typical infrared carbon-hydrogen stretching region for meibum from a 2-year-old Caucasian male. (**B**) Typical phase transition of meibum from a four-year-old Caucasian female. The larger the CH stretching frequency the larger the disorder (fluidity) of the lipid.

Lipid phase transitions were measured for meibum from the young cohort (*My*) and meibum from the older cohort (*Mo*). The frequency of the symmetric CH_2_ stretching band near 2850 cm^−1^ ($\tilde{v}$_sym_) was used to estimate the *trans* to *gauche* rotamer content of the hydrocarbon chains (Figure 1A) [23], and it increased with an increase in temperature (Figure 1B) concurrent with a decrease in intensity [23,27]. The more *gauche* rotamers, the higher the value for $\tilde{v}$_sym_. Two of the phase transition parameters measured in the current study, the minimum and maximum vibrational frequency of the C–H symmetric stretch ($\tilde{v}$_sym_), correspond to the most ordered and disordered states of hydrocarbon chains, respectively. The relative cooperativity of the phase transition describes how the order of a lipid influences that of neighboring lipids. Broad phase transitions have a relatively smaller absolute value of the cooperativity. Lipid order was calculated at 33.4 °C, the temperature at the surface of the eye, and at 36 °C, the temperature of the eye lid [28]. The phase transition parameters are listed in Table 1. The phase transition temperature and lipid order at 33.4 °C and 36 °C, increased significantly with age (from Pearson’s coefficient) for the samples studied, *p* = 0.031. r = 0.342; *p* = 0.032, r = 0.34; *p* = 0.013, r = 0.40, respectively. The same parameters were lower for *Cy* compared with *Co* (Table 1). All of the other phase transition parameters were not significantly different *p* > 0.05 for *Cy* compared with *Co.*

### 2.2. Langmuir Trough Study

**Figure 2:**
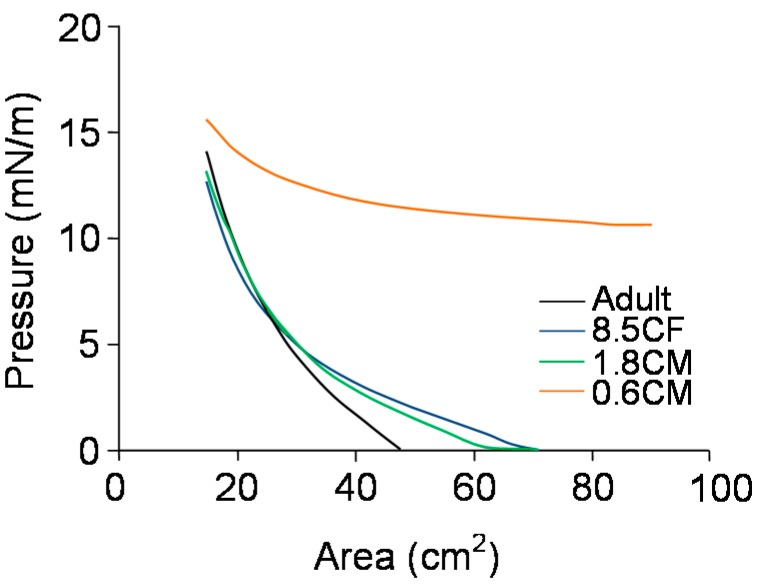
Pressure-area isotherms of meibum films of infants, children, and adults at 35 °C at the air-artificial tear interface.

Parameters measured for the Langmuir trough study are listed in Table 2. Meibum samples upon spreading on AT (artificial tear) solution at maximum area showed a zero baseline spreading pressure, except the infant sample, 0.6CM. The lift-off area, the surface area at which pressure rise over baseline was first observed upon compression, for the adult sample was ~47 cm^2^, while for children samples, 8.5CF and 1.8CM, it was higher, ~68 cm^2^. All pressure-area isotherms showed a slow and continuous increase in pressure upon compression, typical of Meibomian films (Figure 2). The maximum surface pressures at the highest compression (Π_max_) were 14 ± 1 mN/m. The rheology of the infant sample, 0.6CM, was different from the other meibum samples. Its baseline spreading pressure was high, ~10 mN/m, and the pressure upon compression increased slowly over a wide surface area demonstrating a very high compressibility (Figure 2).

**Figure 3:**
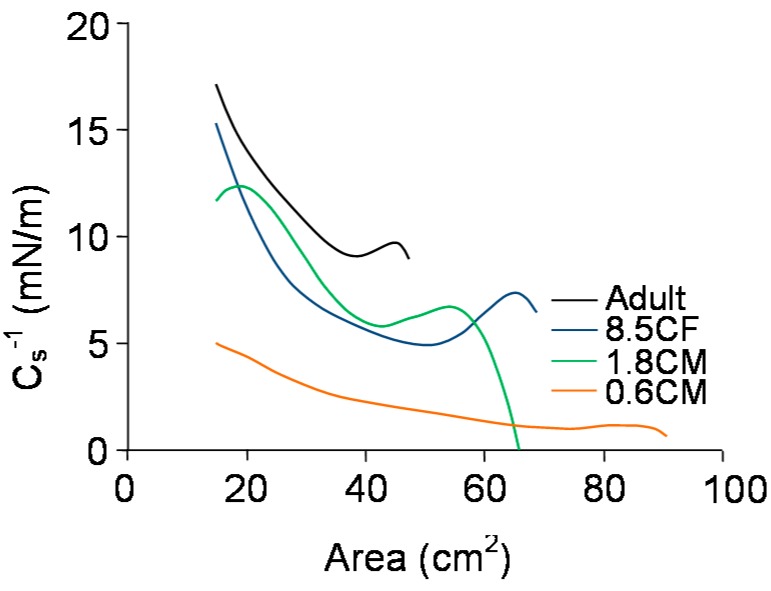
Reciprocal compressibility modulus as a function of surface area for meibum films of infants, children, and adults at 35 °C at the air-artificial tear interface.

The reciprocal compressibility modulus, C_s_^−1^, calculated from the pressure area isotherms, gave information on the compressibility and physical state of the meibum films. A plot of C_s_^−1^ with surface area (Figure 3) showed that C_s_^−1^ for the adult sample had an inflection just after lift-off of 47–38 cm^2^. The pressure then increased steadily and assumed a maximum value of 17 mN/m at the highest compression. Sample 8.5CF started with a lower C_s_^−1^ compared with the adult sample at lift-off, showed a longer infection, 68–49 cm^2^, and assumed a maximum value of 15 mN/m at the highest compression. Sample 1.8CM started with zero C_s_^−1^ at the lift-off, showed two long inflections at 66–43 cm^2^ and 19–15 cm^2^, and assumed a maximum value of 12 mN/m at the highest compression. Sample 0.6CM started with a C_s_^−1^ near zero at the lift-off, increased extremely slowly with compression and assumed a maximum value of 5 mN/m at the highest compression.

**Figure 4:**
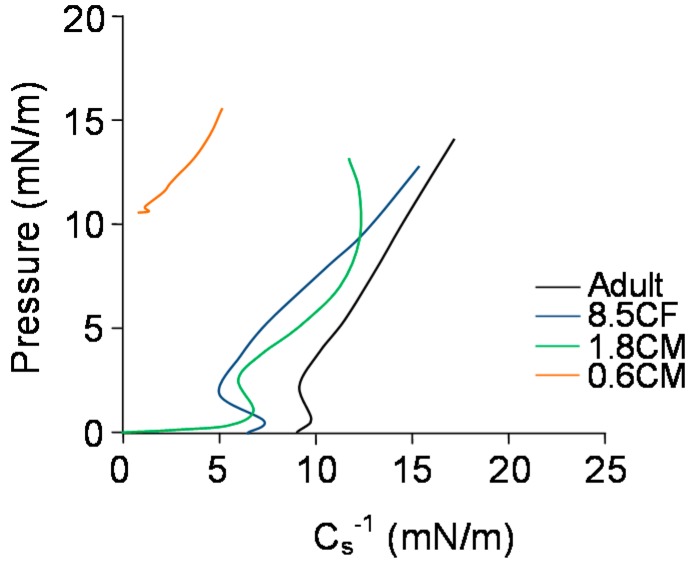
Reciprocal compressibility modulus as a function of surface pressure for meibum films of infants, children, and adults at 35 °C at the air-artificial tear interface.

A plot of C_s_^−1^ with surface pressure (Figure 4) showed that for the adult meibum sample, C_s_^−1^ had an inflection after lift-off at low pressures (0–2 mN/m) and the maximal C_s_^−1^ (17 mN/m) was achieved at Π_max_ of 14 mN/m. Sample 8.5CF showed a lower C_s_^−1^ at lift off, had a longer inflection at similar low pressures and its maximal C_s_^−1^ (15 mN/m) was achieved at a Π_max_ of 13 mN/m. Sample 1.8CM with a zero C_s_^−1^ at lift off and two long inflections at two pressure ranges (lower 0–3 m and higher 10–13 mN/m) achieved its maximal C_s_^−1^ (12 mN/m) at a Π_max_ of 13 mN/m. Sample 0.6CM with near zero C_s_^−1^ at lift off and one inflection at higher pressures (10–11 mN/m) achieved its maximal C_s_^−1^ (5 mN/m) at a Π_max_ of 15 mN/m.

## 3. Discussion

There is a paucity of data on the physical characteristics of meibum from children and babies below eight years of age. Meibum from babies less than four years old have never been studied in detail and the 21 samples below six years of age examined in this study are the highest number studied in this age group to date. 

We observed an increase in the lipid order (stiffness) and the phase transition temperature with age between one and 25 years of age. The tandem increase would be expected as lipid order and the phase transition temperature are directly related [23]. The observed increase in lipid order with age below 25 years of age was not expected as above 25 years of age, lipid order has been shown to decreases slightly but significantly [24,29] The slight decrease in hydrocarbon chain order above 25 years of age does not contribute much to lipid stability because as stated in the Introduction, there is very little change in tear stability above 25 years of age. The increase in lipid order with age below 25 years of age measured in the current study was not in agreement with a study of seven meibum samples three to six years of age [25], which showed that the lipid order of the cohort was much higher than that reported for other ages. The discrepancy was due to two samples, three and four years of age that had an order of 62 and 67%, respectively, much higher than the order of any meibum sample and more than two standard deviation units above the order of aged matched controls. The increase in lipid order and phase transition temperature with age between one and 25 years of age, observed in the current study, may contribute to the observed decrease in tear film stability with age as the largest change in tear film stability occurs below 20 years of age (see Introduction). Correlation does not necessitate cause, but one may speculate that more ordered lipids could cause the tear film lipid layer to become too viscous, a state where deformations become irreversible. Furthermore, too much lipid order may cause lipids on the surface of tears to aggregate into “islands” (lateral phase separated) keeping them from spreading. In support of this idea, lipids from donors with dry eye symptoms and an unstable tear film due to meibomian gland dysfunction (MGD) have the most ordered lipid measured, 47% ordered [23]. Thus, in context to the increase in lipid order with age below 25 years of age and the high order of meibum from donors with MGD, the increase in lipid order with MGD is an exacerbation of aging and could contribute to tear film instability. Too much lipid order may also keep meibum from flowing out of the meibomian glands. We calculated from the current study that the 2- to 3-degree higher temperature of the Meibomian glands compared with the tear film surface [28] would cause the lipids in the meibomian gland to be about 16% less ordered than the lipids on the surface of tears. The difference in order could facilitate the flow of meibum out of the Meibomian glands and facilitate the positive effects of ordered lipids on the surface of tears.

An increase in lipid order driven by saturation causes changes in the rheology of human meibum surface films, resulting in stiffer, more elastic films with lower lift off, increased maximum surface area, and an increase in the maximum compressibility modulus [24,30]. In the current study, except for no difference in the maximum surface area, the differences in the rheology of *Cy* compared with *Co* could be explained by the differences in lipid order between the cohorts [24,30]. As unsaturation has been shown to increase with age [25], one would expect a decrease in lipid order with age, as observed in meibum from donors above 25 years of age, but opposite for meibum from donors below 25 years of age [31]. Other factors that could influence lipid order include cholesteryl esters, hydrocarbon chain length [23], proteins [20,32], and hydrocarbon chain branching. Antieso branching rather than iso branching would be expected to fluidize hydrocarbon chains [33].

Pressure-area isotherms of all meibum samples indicate the existence of a liquid expanded state of interfacial Meibomian films, but a closer look at the nature and variations of reciprocal compressibility modulus within different samples of different ages conveys further information regarding their characteristics and functions that is useful in explaining higher stability in the tear films of children and infants.

The common features of all meibum samples were that they all were highly compressible without any collapse. Lack of collapse and very low molecular area (~10 Å^2^, if an apparent mean molecular weight of about 720 was assigned to meibum [34,35,36,37,38]) at maximum compression is indicative of multilayer formation which happens successively with gradual increase in thickness along the course of film compression. This concurs with the multi-molecular thick tear film lipid layer (about 100 nm or 20 molecules thick) typically dominated by nonpolar components like wax esters and cholesterol esters [39]. Low reciprocal compressibility modulus of all meibum samples correspond to a liquid expanded state [40]. The multilayered structure of meibum facilitates the quick rearrangement of molecules during blinking allowing the tear film lipid layer to withstand the high pressures of blinking. The non-linear dependence of surface pressure on reciprocal compressibility modulus is indicative of the existence of a multicomponent viscoelastic film of Meibomian lipids [41]. The fact that meibum is viscoelastic and its molecular structure governs its viscoelasticity has been substantiated by rheological studies [42].

In comparison with adult meibum, the meibum samples from children and babies had a lower reciprocal compressibility modulus both at low compression and at the highest compression. A highly compressible and reversible multilayered structure may be responsible for the observed low values. Such low reciprocal compressibility modulus accompanied with multilayered organization allows meibum films of children and babies to quickly reorganize and recover from deformation. This makes meibum films of children and babies more stable (more tolerant to high shear stress of blinking) than that of adults. An extremely low value of reciprocal compressibility modulus for meibum from babies shows it is exceptionally stable.

Compared with *Mo*, *My* had a flatter isotherm at high-film areas and higher surface activity, the surface pressure = 10 mN/m at 100% area prior the start of compression. Proteins could account for this observation as a protein enriched layer, but not lipids, is known to form highly compressible films [43]. Proteins are found in meibum [20,43,44,45,46,47] and the meibum protein profile changes dramatically with age [20]. However, the amount of protein measured in the current samples was not measured. Compared with adult meibum, the increased surface activity of an equivalent amount of *My* suggests an elevated concentration of surfactant molecular species in *My*.

The rheology of the infant sample, 0.6CM, was different from the other meibum samples. Given the small sample size and variability inherent in human meibum samples, it would be premature to speculate on the physiological significance of the difference. The Introduction cites studies that show major differences occur in tear film stability between the age of 0.6 years and two years. Future studies may or may not find the difference to be meaningful. Contamination from the trough are unlikely to contribute to the observed difference as cleaning of the trough, as stated in the Methods Section, was thorough. The rheology experiments were repeated three times to ensure reproducibility of the data.

Exceptionally high stability of the interfacial films of meibum from babies can help in explaining low blink rates in infants in comparison with adults. Very low blink rate in infants (<4/min verses 15–30/min in adults) [16,17,18] has been associated with a thicker lipid layer and greater ability to withstand repeated compression and expansion [2,48], thereby showing high elastic resistance to dilational deformation. This can also be correlated to a study where a longer time was needed to stabilize a less viscous tear film, triggering blinking in human subjects [49]. Therefore, adult films requiring more time to stabilize will trigger quicker blinking resulting in higher blink rate while a stable film, like the one in infants, will result in a lower blink rate.

The values of reciprocal compressibility modulus of meibum samples observed in our study are representative of physiological values because we used a physiologically relevant temperature, 35 °C, pH, 7.4, and salt composition (artificial tears). The variations in other studies are likely to be due to different experimental conditions used such as a different meibum source (bovine), lower temperatures, 25 °C or 28 °C, and a non-physiological subphase of saline [36,41] or tris-buffered saline [50]. In particular, temperature is a major factor that influences the behavior of meibum [37]. As temperature rises, lipid films show lower reciprocal compressibility because higher temperature results in more fluid films. Very high reciprocal compressibility modulus observed for bovine meibum [36] or a mixture of synthetic lipids [51] in comparison with human meibum, and variations due to other experimental conditions, highlights the need for having a consensus for conducting experiments under standard conditions, ideally close to physiological conditions [37], across various laboratories to allow effective comparisons. If the composition and physical properties of meibum from people with a more unstable tear film such as adults and people suffering from dry eye could be made to have the physical properties of meibum of babies and infants with a more stable tear film, one could hope that the signs and symptoms of dry eye could be ameliorated. 

## 4. Materials and Methods

### 4.1. Collection and Processing of Human Meibum

Written, informed consent was obtained from all donors or their guardians. Human meibum samples were collected from participants recruited from the Kentucky Lions Eye Center, Louisville Kentucky. Meibomian gland orifices of the participants showed no evidence of keratinization or plugging with turbid or thickened secretions, and no dilated blood vessels were observed on the eyelid margin. The participants did not recall having dry eye symptoms. Protocols and procedures were reviewed by the University of Louisville Institutional Review Board (code: 11.0319, 19 July 2018). All procedures were in accord with the Declaration of Helsinki. Meibomian glands were expressed by lightly compressing the eyelids with strict attention to avoid touching the eyelid margin during expression. All four eyelids were expressed, and approximately 0.5 mg of meibum was collected per individual. The expressate was collected with a platinum spatula and dissolved in a vial of chloroform. None of the samples were pooled.

### 4.2. Infrared Spectroscopic Study

Meibum was collected and lipid phase transitions were measured as described previously [21]. Curves were fit using Sigma plot 10 software (Systat Software, Inc., Chicago, IL, USA) and the confidence levels were obtained from a critical value table of the Pearson product–moment correlation coefficient. Averages were compared using the Student’s t test. A value of *p* < 0.05 was considered statistically significant. Data are reported as the mean plus or minus the standard error.

### 4.3. Langmuir Trough Study

Surface pressure-area profiles of meibum samples were recorded using a computer-controlled single-barrier Langmuir Teflon trough (Nima 102M; Nima Technology Ltd., Coventry, UK) with a surface area of 15 cm^2^–90 cm^2^. The surface pressure was measured by a pressure sensor with a Wilhelmy plate (Whatman, Chr 1 filter paper). The trough was thoroughly cleaned with chloroform before each experiment. It was enclosed in a transparent Perspex cabinet to avoid air currents and airborne contaminants. The temperature of the trough was maintained at 35 °C. The trough was filled with an AT solution [52] which emulated the salt composition of human tears (NaCl 6.6 g/L; KCl 1.7 g/L; NaHCO_3_ 1.4 g/L; CaCl_2_.2H_2_O 0.15 g/L; NaH_2_PO_4_.H_2_O 0.1 g/L; MOPS 4.18 g/L; pH 7.4). Purified water (Milli-Q, resistance >18.2 MΩ; Millipore, Billerica, MA, USA) was used for preparing AT solution. The surface of the AT solution was cleaned with a vacuum aspirator until a clean surface was achieved (pressure change <0.02 mN/m when the surface area was compressed and expanded completely). Lipid sample (20 µL of 1 mg/mL) in chloroform was spread drop-wise on the surface of AT solution using a microsyringe (Hamilton Co., Bonaduz, Switzerland) and chloroform was allowed to evaporate for 10 min. The lipid film was compressed and expanded with a barrier speed of 15 cm^2^/min and changes in pressure (∏) with area (A) were recorded as Π-A isotherms. Experiments were repeated at least three times.

Compressibility (C_s_) of interfacial films at a given surface pressure was calculated from the compression part of Π-A isotherm data using the following equation and was expressed in millinewton/meter (mN/m) [40]:

$$\;C_{s}=-\frac{1}{A_{\Pi }}\;\left(\frac{\mathrm{dA}}{d\Pi }\right)\;$$

where A_Π_ is the area at a surface pressure Π. The inverse of C_s_ was used to determine in-plane (2 dimensional) elasticity modulus, also called reciprocal compressibility modulus or C_s_^−1^ using the following equation and was expressed in millinewton/meter (mN/m):

$$\;C_{s}^{-1}=\frac{1}{C_{s}}\;=\;-A_{\Pi }\;\left(\frac{d\Pi }{\mathrm{dA}}\right)\;$$

The physical state of the interfacial films was determined from C_s_^−1^ values as per Davies and Rideal [40] (<100 mN/m refers to liquid-expanded state, 100–250 mN/m to liquid-condensed state, and >250 to solid state of the film).

## 5. Conclusions

Changes in the lipid phase transition parameters of meibum lipid with dry eye are an exacerbation of the changes observed with age. If lipid order does play a role in tear film stability, and is not just a marker for it, the increase in meibum lipid order from one to 25 years of age may add to the stability of the tear film. Too much order, as observed with meibum from donors with MGD, is likely to decrease tear film stability. The lower reciprocal compressibility moduli of meibum films from children and babies compared with meibum from adults reiterates higher stability in their films which spread better, resist deformation and their ability to be quickly restored after deformation (blinking).

## Figures and Tables

**Table 1:** Donor demographics and phase transition parameters.

Parameter	*Cy*, *My*	*Co*, *Mo*	*p*
Average Age (year)	3.9 (0.5)	20.0 (0.8)	>0.05
Age Range (year)	1 to 12	13 to 25	
Gender (% male)	76	78	
Race (%)	C (88), B (8), A (4)	C (72), B (5.5) A (17) ? (5.5)	
Tm	27 (1)	31.0 (0.8)	0.028 *
Cooperativity (Hill Coefficient)	7.3 (0.6)	7.8 (0.6)	>0.05
Order 36.0 °C (% *trans*)	32 (1)	37 (2)	<0.02 *
Order 33.4 °C (% *trans*)	37 (1)	42 (2)	0.008 *
Δ enthalpy (kcal/mol)	141 (8)	142 (9)	>0.05
Δ entropy (kcal/mol/degree)	0.45 (0.02)	0.48 (0.03)	>0.05
Magnitude (cm^−1^)	4.2 (0.1)	4.0 (0.2)	>0.05
Minimum Frequency (cm^−1^)	2849.60 (0.04)	2849.63 (0.08)	>0.05
Maximum Frequency (cm^−1^)	2853.77 (0.09)	2853.7 (0.2)	>0.05
Δ Order 33.4 °C–36.0 °C (% *trans*)	4.8 (0.7)	5.1 (0.3)	>0.05
Number of Participants	25	18	

*Cy* = younger cohort, *Co* = older cohort, *My* = meibum from *Cy*, *Mo* = meibum from Co, C = Caucasian, B = Black, A = Asian, ? = race unknown. (Standard Deviation). * = significant difference (*p* < 0.05).

**Table 2:** Reciprocal compressibility moduli and inflections for meibum films with maximal reciprocal compressibility moduli ($C_{\mathrm{smax}}^{-1}$) and the surface pressure (Π_max_) at which they are achieved.

Sample *	C_s_^−1^ at Lift-Off (mN/m)	Surface Area Range for Inflection (cm^2^)	Pressure Range for Inflection (mN/m)	$C_{smax}^{-1}\;(\mathrm{mN}/m)$	Π_max_ (mN/m)
Adult	9	47–38	0–2	17	14
8.5CF	6	68–49	0–2	15	13
1.8CM	0	66–43; 19–15	0–3, 10–13	12	13
0.6CM	Near zero	90–72	10–11	5	15

* Numbers represent age (years, months); C = Caucasian; M = male; F = female.
